# Impacts of Water Stress on Forest Recovery and Its Interaction with Canopy Height

**DOI:** 10.3390/ijerph15061257

**Published:** 2018-06-13

**Authors:** Peipei Xu, Tao Zhou, Chuixiang Yi, Hui Luo, Xiang Zhao, Wei Fang, Shan Gao, Xia Liu

**Affiliations:** 1 State Key Laboratory of Earth Surface Processes and Resource Ecology, Faculty of Geographical Science, Beijing Normal University, Beijing 100875, China; xupei@mail.bnu.edu.cn (P.X.); luohui3377@163.com (H.L.); gsapril@163.com (S.G.); 201621480027@mail.bnu.edu.cn (X.L.); 2 Key Laboratory of Environmental Change and Natural Disaster of Ministry of Education, Academy of Disaster Reduction and Emergency Management, Faculty of Geographical Science, Beijing Normal University, Beijing 100875, China; 3 School of Earth and Environment Science, Queens College of the City University of New York, New York, NY 11367, USA; cyi@qc.cuny.edu (C.Y.); Wei.Fang@qc.cuny.edu (W.F.); 4 State Key Laboratory of Remote Sensing Science, Jointly Sponsored by Beijing Normal University and Institute of Remote Sensing and Digital Earth of Chinese Academy of Sciences, Beijing 100875, China; zhaoxiang@bnu.edu.cn

**Keywords:** canopy height, drought, forest recovery, remote sensing, risk assessment

## Abstract

Global climate change is leading to an increase in the frequency, intensity, and duration of drought events, which can affect the functioning of forest ecosystems. Because human activities such as afforestation and forest attributes such as canopy height may exhibit considerable spatial differences, such differences may alter the recovery paths of drought-impacted forests. To accurately assess how climate affects forest recovery, a quantitative evaluation on the effects of forest attributes and their possible interaction with the intensity of water stress is required. Here, forest recovery following extreme drought events was analyzed for Yunnan Province, southwest China. The variation in the recovery of forests with different water availability and canopy heights was quantitatively assessed at the regional scale by using canopy height data based on light detection and ranging (LiDAR) measurements, enhanced vegetation index data, and standardized precipitation evapotranspiration index (SPEI) data. Our results indicated that forest recovery was affected by water availability and canopy height. Based on the enhanced vegetation index measures, shorter trees were more likely to recover than taller ones after drought. Further analyses demonstrated that the effect of canopy height on recovery rates after drought also depends on water availability—the effect of canopy height on recovery diminished as water availability increased after drought. Additional analyses revealed that when the water availability exceeded a threshold (SPEI > 0.85), no significant difference in the recovery was found between short and tall trees (*p* > 0.05). In the context of global climate change, future climate scenarios of RCP2.6 and RCP8.5 showed more frequent water stress in Yunnan by the end of the 21st century. In summary, our results indicated that canopy height casts an important influence on forest recovery and tall trees have greater vulnerability and risk to dieback and mortality from drought. These results may have broad implications for policies and practices of forest management.

## 1. Introduction

Forest ecosystems are an important carbon sink and play a significant role in the global carbon cycle [1]. In the context of ongoing global climate change, the frequency and intensity of drought are increasing [2], which has a substantial impact on the structure and function of many forest ecosystems [3]. Drought stress not only affects the vigor and growth of individual trees, but also may hasten tree death and forest degradation [3], resulting in reductions of forest productivity [4]. Therefore, to better adapt to and alleviate the impacts of climate change on forest ecosystems, it is necessary to correctly evaluate the characteristics of forest response to drought [5,6].

Accurate assessments of drought in forests should include two aspects: (1) showing the current impact of water stress based on forest growth variation, as indicated via changes in the vegetation index from remote sensing observation [7] and tree rings from cores [8]; (2) evaluating the forest recovery potential according to the recovery path of forest growth as water availability improves [9]. The characteristics of forest response to water stress show an obvious gradualness, accumulation, and mutability. Usually, a forest is greatly affected when local water stress exceeds a biologically relevant threshold [8], which would generate substantially different characteristics compared to the predisturbed condition. However, the effect of drought is often determined by interactions among multiple factors [10]. Drought events may cause forest decline and tree mortality a few years later due to time-lag effects [11]. Therefore, by studying the characteristics of forest recovery after drought events, we can predict the drought response of forests in a more accurate way.

The climate is the most important environmental factor affecting tree growth in forest ecosystems [12,13]. Water availability improves following a drought event, which will influence forest recovery in the short term and may further affect temperature and precipitation regimes caused by global climate changes [9]. Studies based on the historical reconstruction of tree rings have shown that increases in water availability were associated with higher ring widths [12,13], and that decreases in water availability, accompanied by heat and drought events, would lead to reduced tree growth [10].

However, for forests with differing structures, their water requirements vary widely due to varying forest types, ages, canopy heights, and tree trunk sizes [14,15]. Such differential attributes should be considered when trying to reveal and understand the relationship between water availability and forest growth. Compared to short trees, the path and height of internal water transportation are longer and higher in tall trees, so the effect of gravity and path-related resistance is also greater, resulting in their substantial sensitivity to changes in water [15,16,17]. As such, at the global scale, tall trees are more sensitive than short trees to drought stress [18]. However, empirical studies of how forest attributes such as canopy height affect forest recovery, and how they interact with the post-drought improvement of water availability, are still limited. Considering the great range in differences of forest attributes caused by human activities—such as deforestation and afforestation—and natural disturbances [19], it is crucial to investigate the potential impacts of canopy height on forest recovery after drought. 

Currently, three methods are typically used to explore the interaction between climate and forest attributes. The first one is the controlled experiment method, which analyzes the relationship between forest growth and forest attributes in the field [14,15]. The second one is using time-series data of natural vegetation growth dynamics, for example, those from ground investigations [9] and remote sensing observations [19], which depict the relationship between forest growth and water availability to indirectly reveal the effect of forest attributes. The third one analyzes typical drought cases occurring at the regional scale. In this approach, according to the observed dynamic changes and spatial differences of actual forest disturbance and recoveries, the quantitative relationship between improved water availability and forest recovery is investigated [9] and the influence from forest attributes such as canopy height is revealed.

At the regional scale, combining remote sensing data and typical drought cases is the key way to effectively approach this complex topic [7,20]. Green vegetation strongly absorbs radiation in the red wavelength but strongly reflects it in the near-infrared; for this reason, various vegetation indices calculated by the reflectivity of red and near-infrared wavelengths are widely used in drought studies [8]. For example, remote sensing observations indicated that the prolonged and devastating drought in southwest China during 2009–2013 led to the decline and death of forests, but the drought response varied among forest types [19]. Because the time series of remote sensing observations can provide continuous information on forest dynamics, both temporally and spatially, it establishes a baseline for investigating the effects of forest attributes on post-drought recovery. Another approach, light detection and ranging (LiDAR) technology, can estimate the vertical parameters of a forest to obtain the spatial distribution of its canopy height by inversion [21], which provides strong data support for studying the impact of canopy height on forest recovery following drought events at the regional scale.

Here, we studied how the forest was affected by the extreme drought event (2009–2013) in Yunnan Province, China. Using the enhanced vegetation index (EVI) from remote sensing, the canopy height data from LiDAR, and the standardized precipitation evapotranspiration index (SPEI) data, we quantitatively evaluated the relationships among forest recovery, water availability, and canopy height. We also revealed the variation in water requirements of forests with differing canopy heights during the recovery phase, which provides a reference for more accurate assessments of forest response to future climate change. The objectives of this study were to address two scientific questions: (1) How do canopy heights affect forest recovery after drought? (2) Do canopy heights consistently affect forest recovery under different water stress conditions?

## 2. Materials and Methods

### 2.1. Study Area

**Figure 1:**
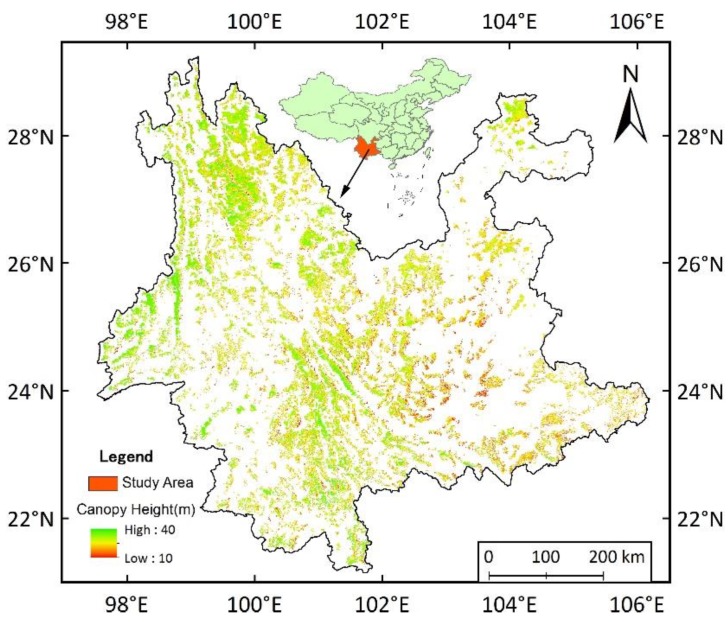
The distribution and canopy heights of forested area in Yunnan Province, southwest China, at a resolution of 1 km [21]. The white region represents the nonforest area.

The study area is located in Yunnan Province, southwest China (Figure 1), which has a total area of 390,000 km^2^ (97.51°–106.18° E, 21.13°–29.25° N). The average annual temperature is 16 °C, the annual precipitation is 1105 mm, and the peak season for plant growing is in July and August [19]. In recent years, multiple severe drought events have occurred in Yunnan. The most prolonged drought event happened between 2009 and 2013, which had a substantial impact on the local forest ecosystems. According to the Eighth National Forest Inventory (http://211.167.243.162:8085/8/index.html), Yunnan has rich forest resources, including a forest area coverage of 50.03%, a forest area of 19,140,000 hectares, and a timber storage volume of 1.69 billion m^3^. The main forest types include mixed forest and various forests dominated by *Pinus* sp., *Quercus* sp., *Eucalypus* sp., and *Abies* sp. [19]. Yunnan is an ideal area to study forest recovery after drought due to its rich forest resources and distinct drought events.

### 2.2. Data and Methods

#### 2.2.1. Data

Remote sensing data: Two major types of remote sensing data were used in this study. One was MODIS EVI (MOD13A1) (http://modis.gsfc.nasa.gov/). EVI served as an indicator of forest growth. Considering that forests are sensitive to water availability during the growing season [8,19], the average EVI from the 193rd to 241st calendar day, corresponding to the growing season of July and August, was calculated to indicate the annual growth of the forest from 2001 to 2014. The other form of data was the spatial pattern of canopy height from LiDAR [21], which was downloaded from the global canopy height distribution map with the resolution of 1 km (https://landscape.jpl.nasa.gov). This map provided estimated mean canopy heights across the land surface from LiDAR rather than from field observations. It provides some of the best canopy height data currently available at regional scales, and prior studies have indicated the canopy heights from this dataset is well correlated with height data from field observations made in Yunnan and southwest China [22].

Forest map: The forest thematic maps of Yunnan Province were digitized and spatially registered from the Seventh and Eighth National Forest Inventory in China [23,24], with a combination of high-resolution remote sensing images and field observations.

Drought index: The SPEI served as an indicator for the intensity of drought stress [25] and water availability during forest recovery. The SPEI data were obtained from the global SPEI dataset (http://sac.csic.es/spei/database.html), which is based on monthly totals of precipitation and potential evapotranspiration collected by the Climatic Research Unit (CRU) of the University of East Anglia [26]. This data had a 0.5 degree spatial resolution and a monthly temporal resolution. To match the time requirement of vegetation growth, this study used SPEI in July with a 12-month timescale [20]. Water condition could be divided into nine grades based on SPEI values [27,28] (Table S1). The threshold value of SPEI for a “dry condition” was −0.5.

Climate scenario data: To assess the change in drought intensity in the context of future climate change, two projected scenarios, RCP2.6 and RCP8.5, were used. Moreover, the corresponding SPEI data were calculated to analyze their potential impacts on forest growth. The projection data were obtained from CMIP5, which includes four climate models from the United States, France, Japan, and Norway: GFDL-ESM2M [29], IPSL-CM5A-LR [30], MIROC-ESM-CHEM [31], and NorESM1-M [32]. That dataset contained daily temperature and precipitation from 1951 to 2099 at the spatial resolution of 0.5 degree. Before 2005, the simulations were carried out based on actual greenhouse gas emissions, but multiple different emission scenarios were considered for simulations after 2005. In our study, the lowest (RCP2.6) and highest (RCP8.5) emission scenarios were considered as well as major climatic factors, such as monthly average temperature and precipitation, and the calculated SPEI [25].

#### 2.2.2. Statistical Model of Canopy Height and Optimum Water Requirement

As forests with different canopy heights have different water requirements [33], the forest response to climate change should depend partly on its canopy height. The long time series of satellite observations measure vegetation growth (EVI) with spatiotemporal continuity. Combined with the SPEI data, it allowed us to estimate the optimum water condition (${\mathrm{SPEI}}_{OP}$) for growth at forests with different canopy heights. Under optimal water availability, forests usually exhibit their optimum growth [34] with a maximum EVI (${\mathrm{EVI}}_{max}$). Therefore, the relationship between the canopy height and optimum water requirement may be quantified through a linear regression model of ${\mathrm{SPEI}}_{OP}$ (SPEI at ${\mathrm{EVI}}_{max}$) over canopy heights.

In the present study, the ${\mathrm{EVI}}_{max}$ of all the forest pixels in Yunnan from 2001 to 2014 were obtained, and the corresponding ${\mathrm{SPEI}}_{OP}$ was selected when ${\mathrm{EVI}}_{max}$ occurred. This ${\mathrm{SPEI}}_{OP}$ showed the water availability under which forests reach their maximum growth potential in nature. Next, mean ${\mathrm{SPEI}}_{OP}$ were calculated for pixels with the same canopy height, and a linear regression model was established between ${\mathrm{SPEI}}_{OP}$ and canopy height to reflect their relationship. In order to get valid estimates of mean ${\mathrm{SPEI}}_{OP}$, the number of pixels for each category of canopy height had to be at least 1% of the total pixel numbers to be included in the regression model.

#### 2.2.3. Selecting Forest Recovery Area

To reveal the relationships among forest recovery, water availability, and canopy height, the forest area chosen should satisfy two criteria simultaneously: (1) the impact of drought on the forest was significant during the event (2009–2013); and (2) water availability was restored to a nondrought condition in 2014.

(1) Determination of Forest Area Significantly Affected by Drought

According to Yunnan’s forestry survey (based on a combination of high-resolution satellite imageries and random sampling in the field), 1.88 million hectares of forest area were severely damaged during 2009–2012, accounting for 10% of the total forested area in Yunnan [19]. Therefore, 10% of the forested area in Yunnan Province was selected as the drought-affected area for this study, and the detailed spatial distribution of this affected area was determined by the EVI deficiency index (ED) (Equation (1)). The forest exhibited optimum growth under no drought stress, yielding a maximum level of EVI (${\mathrm{EVI}}_{max}$), while exhibiting hindered growth with drought stress, yielding a less-than-optimum EVI (${\mathrm{EVI}}_{i}$). The stronger the stress was, the greater was the deviation between EVI and ${\mathrm{EVI}}_{max}$. In our study, the ${\mathrm{EVI}}_{max}$ of each grid during 2001–2014 was first obtained, and ED for each grid was then calculated with Equation (1). Finally, the ED values were sorted, and the top 10% of forests with the most negative ED (i.e., the most relative EVI reduction) were selected as areas being affected by drought.

(1) $${\mathrm{ED}}_{i}\left(\%\right)=\frac{{\mathrm{EVI}}_{i}-{\mathrm{EVI}}_{max}}{{\mathrm{EVI}}_{max}}\times 100$$

where ${\mathrm{ED}}_{i}$ and ${\mathrm{EVI}}_{i}$ indicate the ED and EVI at the ith year during the 2001–2014 period.

(2) Determination of Potential Forest Recovery Areas after Drought

The areas of potential forest recovery were determined by the SPEI. SPEI data showed that the water availability in Yunnan Province had improved considerably in 2014. According to the drought classification (i.e., grades of water condition mentioned earlier) of SPEI, ~80% of the region was considered as being nondrought-affected areas (SPEI > −0.5). Therefore, for our study, we selected the drought-affected areas during 2009–2013 from these nondrought-affected areas in 2014 to serve as the potential forest recovery area. We used the increase of ED and EVI as indicators to analyze the relationship between forest recovery and canopy height from both absolute and relative growth of the forest.

### 2.3. Statistical Analysis of the Difference in the Recovery

#### 2.3.1. Relationship between Forest Recovery and Canopy Height

To study the dependence of forest recovery on canopy height, their correlation was explored by a linear regression model. Forest recovery areas were determined based on forest survey data collected in 2012; so, 2012 was selected as the baseline to calculate the increases of EVI and ED in 2014. A linear regression model between the ED (EVI) increase and canopy height was established. Specifically, we first calculated the difference of ED (EVI) in the forest potential recovery area between 2012 and 2014; that is, ED (EVI) in the year following the drought event (2014) was subtracted by the ED (EVI) of the year during drought (2012). A greater difference represented a higher degree of recovery. Finally, the average increases of ED (EVI) were calculated among pixels with the same canopy heights. Linear regression models of average ED (EVI) increases against canopy heights were established, indicating the quantitative relationship between forest recovery and its canopy height.

#### 2.3.2. Relationship between Forest Recovery and Water Availability

Similar to the spatial heterogeneity of canopy height, a significant heterogeneity in water availability across the studied area was also observed in Yunnan. Both may affect forest recovery in Yunnan after the drought (2014). In our study, we analyzed the relationship between forest recovery and canopy height under various levels of water conditions. Several linear regression models between the ED (EVI) increases and canopy height were established to obtain the determination coefficient (*R*^2^) under various water availability conditions. The recovery area was then divided into three subareas based on the SPEI in 2014: normal condition (−0.5 < SPEI ≤ 0.5), mild wetness (0.5 < SPEI ≤ 1), and moderate wetness (1 < SPEI ≤ 1.5). Finally, the difference in the regression models between height and the ED (EVI) increases was compared under various water availability conditions.

To quantify the influence of water availability on the relationship between forest recovery and canopy height, we analyzed the difference in their correlation intensity under various water availability conditions, with SPEI values spanning from the minimum value of nondrought (−0.5) to the near maximum value (1.0). One hundred a fifty ${\mathrm{SPEI}}_{j}$ were selected (${\mathrm{SPEI}}_{j}$ = −0.5, −0.49, −0.48, …, 1, the interval is ΔSPEI = 0.01). After that, for each ${\mathrm{SPEI}}_{j}$, the coefficient of determination $R_{j}^{2}$ for the regression between the ED (EVI) increases and height was calculated in the recovery areas of 2014 with $\mathrm{SPEI}>{\mathrm{SPEI}}_{j}$, from which 150 values of $R_{j}^{2}$ were obtained. A greater $R_{j}^{2}$ indicated a stronger contribution by canopy height for explaining the variance in forest recovery. Finally, a linear regression model was established for $R_{j}^{2}$ and ${\mathrm{SPEI}}_{j}$ to quantitatively reveal the effect of water availability on the relationship between forest recovery and canopy height. When the water improvement was substantial enough to reach the threshold of ${\mathrm{SPEI}}_{th}$, the regression equation between the ED (EVI) increases and canopy height was no longer statistically significant (*p* > 0.05), suggesting that canopy height no longer impacted the forest recovery.

To eliminate the possible effect of ΔSPEI, nine different ΔSPEI (0.01, 0.02, 0.03.....0.08, 0.09) were selected to calculate nine ${\mathrm{SPEI}}_{th}$, the average of which was designated as ${\mathrm{SPEI}}_{th}$. For example, if the interval was ΔSPEI = 0.02, 75 ${\mathrm{SPEI}}_{j}$ were selected (${\mathrm{SPEI}}_{j}$ = −0.5, ‒0.48, −0.46, …, 1.00). If the interval was ΔSPEI = 0.09, 17 ${\mathrm{SPEI}}_{j}$ were selected (${\mathrm{SPEI}}_{j}$ = −0.5, −0.41, −0.32, …, 1.00). The following steps were the same as mentioned above.

In the context of future climate change, the frequency and duration of droughts may increase and so might their effects on forest. This study evaluated the risk of drought on the forest in the future with the following two indicators. The first was drought frequency. The second one was the frequency of persistent water stress. Continuous water stress not only drives drought’s instant effect on forests, but also affects the recovery of forests in the later phases. Therefore, the frequency of postdrought water stress was also taken into account in our study. To do this, the SPEI values during 1951–2099 in Yunnan were estimated based on the monthly temperature and precipitation under the emission scenarios of RCP2.6 and RCP8.5. In addition, the drought (SPEI < −0.5) frequency before and after 2005, as well as the frequency of incomplete forest recovery due to the insufficient water in the following year—that is, SPEI < −0.5 in the first year and $\mathrm{SPEI}<{\mathrm{SPEI}}_{th}$ in the second year—were determined. Finally, a regional average was calculated to obtain the average frequency of drought and persistent water stress in Yunnan historically (1951–2005) and in the future (2006–2099).

All regression analyses were conducted in EXCEL (Microsoft Office 2013, Microsoft Corporation, Washington, DC, USA). All graphs were made in IDL8.5 and Arcgis10.0 (Environmental Systems Research Institute, Redlands, CA, USA).

## 3. Results

### 3.1. Effects of Canopy Height on the Water Requirements of the Forest

**Figure 2:**
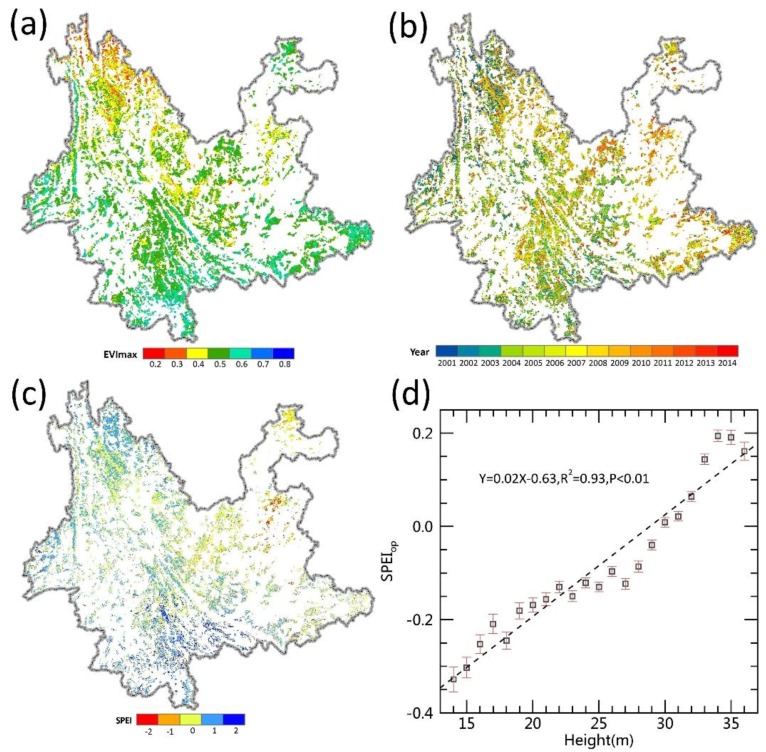
Water requirements of forest vary with canopy heights. (**a**) The maximum enhanced vegetation index (EVI) of forests in Yunnan during 2001–2014; (**b**) The occurrence time of the maximum EVI in (**a**); (**c**) The standardized precipitation evapotranspiration index (SPEI) value at the occurrence time of the maximum EVI (${\mathrm{SPEI}}_{op}$ , which represents the optimal water availability for maximum growth potential; (**d**) Regression of mean ${\mathrm{SPEI}}_{op}$ against forest canopy height (mean ${\mathrm{SPEI}}_{op}$ were calculated for pixels with the same canopy heights). The white regions in (**a**–**c**) represent nonforest areas.

To study how climate and forest attributes interacted to affect forest growth, we used the long time-series data from remote sensing observations to regress optimum water availability (${\mathrm{SPEI}}_{op}$) against canopy height when the forest reached its maximum growth potential (i.e., $\mathrm{EVI}={\mathrm{EVI}}_{max},\;\mathrm{ED}=0$) (Figure 2). A strong positive linear relationship was found (*R*^2^ = 0.93, *p* < 0.01, Figure 2d) showing greater ${\mathrm{SPEI}}_{op}$ in a forest with taller trees. This suggested more water was needed to reach the maximum tree growth as canopy height increased. Taller trees are more susceptible to drought [15,18,35], which may drive variation in forest recovery across space and time.

### 3.2. Forest Recovery Area

**Figure 3:**
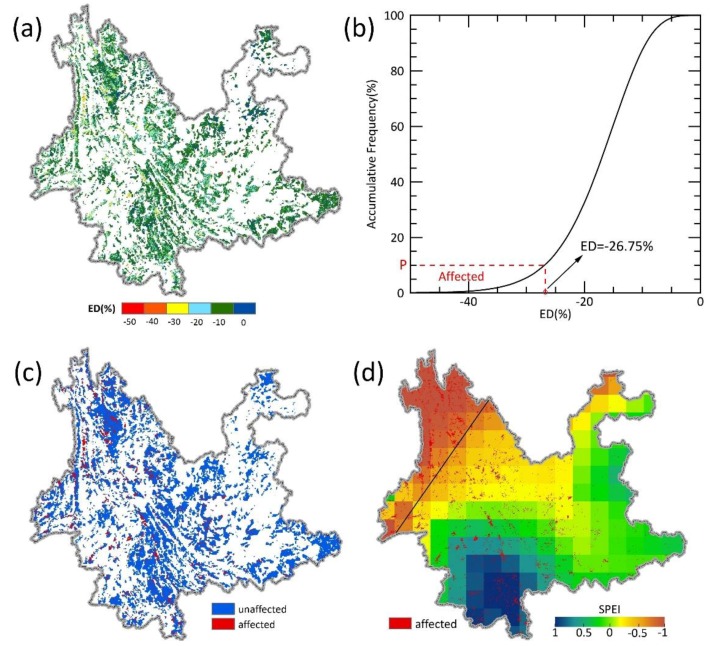
The determination of forest recovery area: (**a**) Average ED (EVI Deficit (%); EVI: enhanced vegetation index) of forested areas during the drought event of 2009–2012 in Yunnan Province. (**b**) The top 10% area with the most negative ED (<−26.75%) was selected as the drought-affected area for this study. (**c**) Forested area in Yunnan was further divided into two regions: affected (ED < −26.75%, red) vs. unaffected (ED ≥ −26.75%, blue). (**d**) Standardized precipitation evapotranspiration index (SPEI) of Yunnan Province in 2014. Forest areas affected by the prolonged drought during 2009–2013 (ED < −26.75%, red spots in (**c**,**d**)) with water condition improved in 2014 (SPEI > −0.5, red spots below the black line in (**d**)) were defined as the potential recovery area of the forest. The white regions in (**a**,**b**) represent nonforest land.

To reveal the effect of canopy height on forest recovery, it is necessary to select the forests affected by drought to analyze their recovery status with improved water availability. There was a dry–wet alternation in Yunnan during 2001–2014 according to the SPEI data (Figure S1). Based on the survey data of the forest disasters and the variation in ED detected by remote sensing during the same period, the corresponding ED range for the affected 10% of the forests was found to be ED < −26.65%. In 2014, the water availability of ca. 80% of the study area converted to nondrought status (Figure 3). These areas were mainly located in the center and south of Yunnan and ca. 95.89% of the recovery area was natural, mostly composed of broadleaf forests (ca. 68.2%) and needleleaf forests (ca. 24%).

**Figure 4:**
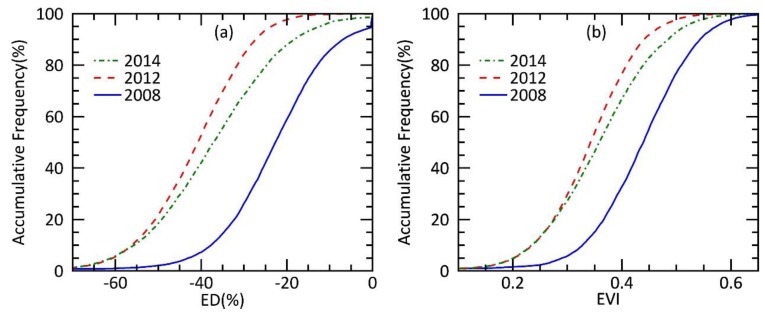
EVI Deficit (ED) (**a**) and enhanced vegetation index (EVI) (**b**) in the recovery areas, affected by drought during 2009–2013 and with improved water condition in 2014, before (blue, 2008), during (red, 2012), and after drought stress (green, 2014).

The cumulative frequency of ED (EVI) before (2008), during (2012), and after (2014) the drought (Figure 4) showed that the distribution of the forest vegetation index shifted significantly to a low value during the drought (2012) compared to before it (2008); that is, both EVI and ED were reduced, suggesting that drought led to a decrease in forest growth. In 2014, both EVI and ED are higher than that in the drought period but lower than in predrought, thus suggesting that the forest growth potential recovered with the postdrought water improvement, but still far from its predrought status.

### 3.3. Impacts of Water Stress and Canopy Height on Forest Recovery

Forest growth was able to recover after the drought with enhanced water availability. To study the effect of canopy height on forest recovery, ED (EVI) increases were grouped by pixels with the same canopy height, and ED (EVI) increases were regressed against canopy height. This yielded a strong negative linear relationship (Table 1 and Figure S2). When the regression was conducted separately for different forest types, the strong negative relationships remained and were consistent across forest types (Table 1). The ED and EVI increases were reduced with greater canopy height. These results suggested that for the forest affected by drought, the recovery was reduced by canopy height.

**Figure 5:**
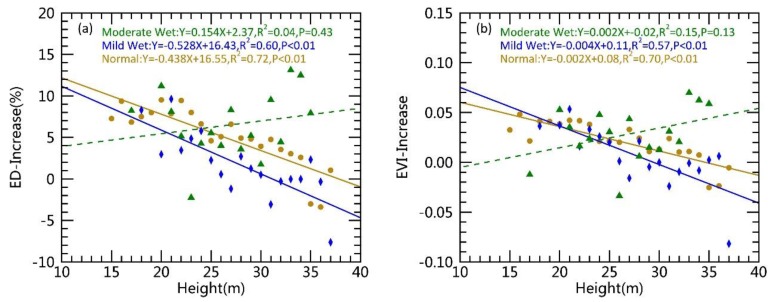
Linear regressions between the increase of EVI Deficit (ED) (from 2012 to 2014) and canopy height (**a**), and between enhanced vegetation index (EVI) (from 2012 to 2014) and canopy height (**b**) under three different water conditions (Normal: −0.5 < SPEI ≤ 0.5; Mild Wet: 0.5 < SPEI ≤ 1; Moderate Wet: 1 < SPEI ≤ 1.5). The effects of canopy heights on forest recovery were no longer significant for the moderately wet condition.

Although the relationship between an ED (EVI) increase and canopy height was revealed, it did not eliminate the effect of moisture spatial variation on forest recovery, if considering the spatial heterogeneity of the nondrought conditions at the regional scale. To further reduce this effect of spatial variation in water availability, they were divided into three grades based on SPEI. Comparing the regression equations between canopy height and ED (EVI) increases under various water availability conditions (Figure 5) showed a strong negative linear relationship (*p* < 0.01) under normal and mild wetness conditions; for these, the ED (EVI) increase was substantially reduced by greater canopy height. For the forests affected by drought, their recovery decreased with the increase of canopy height under all three water availability grades. In addition, *R*^2^ of the regression equation exhibited a decreasing trend under the conditions of normal, mild wetness, and moderate wetness. This suggested that the relationship between recovery and height weakened as water availability improved. Under the moderate wetness condition, no significant linear relationship was observed between the increment of ED (EVI) and canopy height, indicating that recovery was not related to height. Canopy height will not lead to a variation between the recovery of tall and short trees.

**Figure 6:**
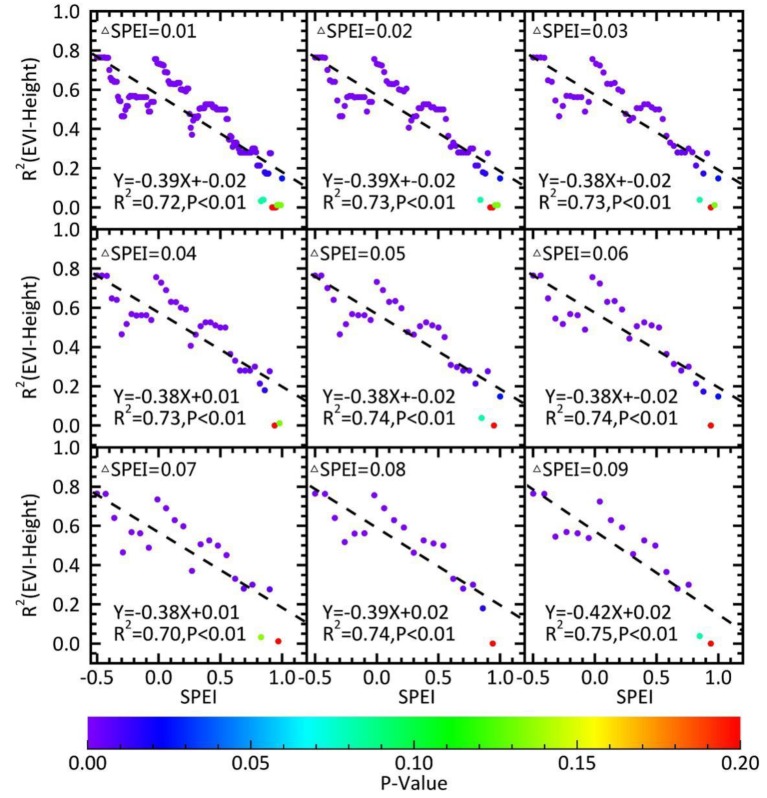
The relationship between forest recovery and canopy height under various water availability. The *x*-axis (SPEI, standardized precipitation evapotranspiration index) represents water availability, and the *y*-axis indicates the determination coefficients (*R*^2^) of the regression equation between the increase of enhanced vegetation index (EVI) from 2012 to 2014 and canopy height. The color-bar indicates the significance of the regression equation. With improved water availability, the relationship between forest recovery and canopy height weakened, suggesting that height is an important factor affecting forest recovery, but its effect depends on water availability and will disappear with sufficient water. The nine panels (with varying delta SPEI) showed a consistent pattern.

Water availability will constrain the effect of height on forest recovery. Better water availability leads to smaller impacts, so theoretically there should be water threshold (${\mathrm{SPEI}}_{th}$). If the water availability is below this threshold, a significant difference in the recovery between tall and short trees is present, whereas no such difference will be observed if it is greater than this threshold. To explore this aspect, the regression *R*^2^ values of ED (EVI) increases and canopy height were calculated under various water availability conditions. In addition, the critical point of the regression at the statistical significance level of *p* = 0.05 was determined (${\mathrm{SPEI}}_{th}$). The results of ED and EVI were consistent from the independent calculations (Figure 6 and Figure S3), and the average ${\mathrm{SPEI}}_{th}$ was 0.85 (SD = 0.02) using the nine ΔSPEI. When the water condition was lower than the critical point (SPEI < 0.85), both EVI and ED increases were significantly negatively correlated to height, and a substantial difference in recovery was evident between the tall and short trees. When the water availability reached the critical point (SPEI > 0.85), the increase of ED (EVI) and height exhibited no significant relationship (*p* > 0.05), and the recovery of tall and short trees was similar.

**Figure 7:**
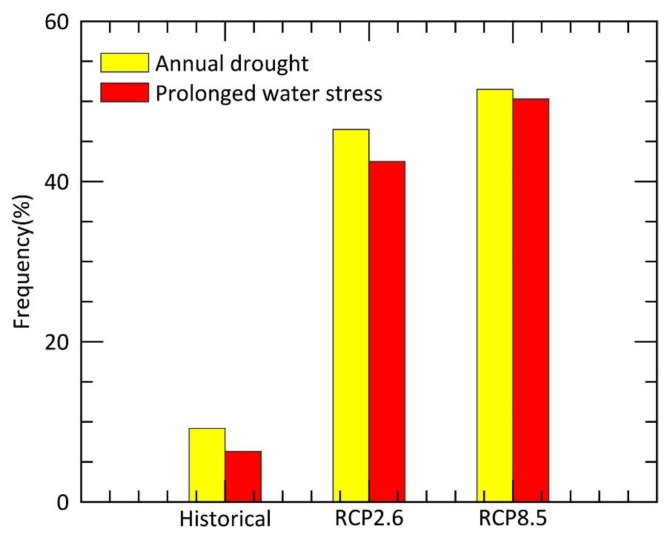
Compared to the historical data (1951–2005), the frequency of drought and prolonged water stress events will increase in Yunnan under the RCP2.6 and RCP8.5 projection scenarios (2006–2099).

In the present study, we calculated the frequency of water stress in the experimental area based on projection scenarios to assess the risk of drought in the future. Our results illustrated that the average frequency of droughts in Yunnan is about 9.2% from 1951 to 2005, while the recovery frequency affected by persistent water stress was 6.3%. For 2006–2099, the predicted average frequencies of droughts in Yunnan were 46.5% and 51.5% under the projection scenarios of RCP2.6 and RCP8.5, respectively, and the recovery frequencies affected by persistent water stress were 42.5% and 50.3% (Figure 7). Therefore, regardless of the carbon emission scenarios, the frequency of prolonged water stress in Yunnan will increase significantly, indicating that controlling carbon emissions will not be very effective in reducing the risk of water stress. The forest in the northern part of Yunnan where trees were taller than in the south will be more susceptible to increasing drought frequency and intensity. As the response to water stress also depends on canopy height according to our results, adjusting the height during forest management may help minimize the drought risk in response to climate change [36].

## 4. Discussion

### 4.1. Forest Recovery Influence by Canopy Height and Water Stress

Canopy height is an important attribute of forest ecosystems [37,38]. It has a significant impact on drought responses of forests [37,38], which can be reflected by multiple potential mechanisms [39]. Some studies demonstrated that soil water is less available to the shallow root systems of small trees, resulting in weaker drought tolerance [40]. Other studies indicated that drought has a greater impact on large trees than small trees [14,15,18], as large trees have a higher water demand for growth. Due to the time hysteresis [41] and the accumulative effect [8,42] of forest responses to the climate, the effect of tree size on forest recovery following drought should be explored. Here, remote sensing data illustrated that the natural forests in Yunnan were recovering as water conditions improved after the drought. Our results suggested that shorter trees recovered more than taller trees in both broadleaf and needleleaf forests when the water availability did not meet the demand of tall trees (Table 1). This is probably caused by higher water demand in the large trees for retaining metabolism and growth. The vertical water conduction path from root to canopy is also longer for large trees, requiring a greater pulling force with a greater moisture gradient [15]. Yunnan experienced a drought with long duration and high intensity during 2009–2013 [19], resulting in the depletion of deep soil water. Therefore, tall trees could not absorb enough soil water even with a more developed root system. However, such influence of canopy height on recovery diminished once water availability was enhanced to certain level (Figure 5 and Figure 6). When we grouped SPEI level into three categories (normal: −0.5–0.5; mild wet: 0.5–1.0; moderate wet: 1.0–1.5), the influence of canopy height on recovery was no longer there for the moderate wet level. Our further analysis revealed that, when the water condition surpassed the threshold of SPEI = 0.85, the pulling force was sufficient enough to transport water from the root to the canopy of all sizes, yielding no difference in recovery between short and tall trees.

The data used in this study included a national forest inventory, satellite observations, and LiDAR inversion. The spatial resolutions of different datasets were not exactly the same, so deviation in the results was expected. Although the canopy height data with 1 km spatial resolution used here was one of the best descriptions of forest vertical structure currently available at regional scales [21,22], its resolution still could not reflect the characteristics of drought response at the tree-level scale. Further division of the pixels demanded for high-resolution remote sensing data (e.g., Quickbird image of 0.6 m) is difficult to apply at the regional scale, such as a whole province in this study. As a result, this study focused on the regional scale and some uncertainties at tree-level were unavoidable. In addition, it was difficult to directly quantify the effect of topographical variables (such as slope, aspect, etc.) on such a large scale, so we addressed the issue indirectly. The effect of topographical variables can be reflected in the EVI [34]. Therefore, we used the standardized index ED instead of EVI as an indicator of tree growth to eliminate or reduce the influence of topography. Nevertheless, all the factors mentioned above may still contribute to the uncertainty of the results.

### 4.2. Implications

Humans have continuously interacted with forest systems [43]. Humans depend on forests for survival and development, from the air people breathe to the wood people use [44,45]. Forests also offer watershed protection, prevent soil erosion, and mitigate climate change [46,47,48]. People in Yunnan strongly depend on forests for their livelihood—food, timber, medicine, tourism, and preserving water resources for hydroenergy generation. The ecological services provided by forests are disproportionally more important when this normally very wet region is going through a severe and prolonged drought (e.g., 2009–2012 in Yunnan). On the other hand, human activities such as deforestation and afforestation have also altered the structure and functions of forests [49]. Drought is one of the greatest threats to forest ecosystems, which can influence productivity and further affect the carbon sink function of forest ecosystems [4]. The IPCC5 report showed that the frequency and duration of drought would increase worldwide in the future [50]. As climate change becomes unavoidable, more attention and effort are called for reducing the impact of drought on forest ecosystems. Our results showed that forest responses to climate were also affected by tree size [51,52,53], which sheds new light on managing forests under the changing climate. Although the economic benefits and ecosystem services of trees increase with their size over time, the risk of mortality or dieback under drought stress also increases with time. Humans might reduce the impact of climate change on forests by manipulating the forest structure, specifically by removing tall trees that have passed their prime time of carbon fixation with additional risks of mortality or dieback during drought and filling the gaps with planting or natural regeneration. Meanwhile, we should also be aware of the various ecological drawbacks of removing large trees, e.g., loss of habitats for wildlife species, loss of structural diversity, and potential nutrient loss [51]. Therefore, we could experiment with a number of silvicultural techniques to explore how different forest ecosystems perform under direr future conditions. We should also assess the effect of structural complexity (the combination of small and large trees) which may cause an improved exploitation of soils and water resources. The practice of actively managing the canopy heights, based on the results of this study, will not only bring economic benefits (i.e., timber and fuels), but also enhance ecosystem services (i.e., revitalizing the forest ecosystems and boosting their net primary productivities). The future scenario data used in this study also showed that frequency of water stress will increase in Yunnan. Although the intensity of drought may be reduced by controlling carbon emissions (Figure S4), the frequency of drought could not be reduced effectively. Forests with large trees will face a higher risk of death and degradation with future warming and a drying climate. Therefore, the effects of canopy height on drought-related risks need to be taken into consideration for policy making and resource management for the effects of climate change on future forests under climate change [36,52].

## 5. Conclusions

Natural and human disturbances significantly alter the structure and function of forest ecosystems, thus affecting their response characteristics and recovery paths under water stress. Revealing the differences in the recovery of forests with varying in canopy heights is the basis for an accurate assessment of the impact of climate change on forest ecosystems. In the present study, we combined remote sensing data (EVI) and meteorological drought index (SPEI) data to analyze the relationship between forest recovery and water availability at the regional level, as well as the variation in the recovery among forests with diverse canopy heights. With improved water availability following drought, the forest in Yunnan was able to recover. This recovery was not only dependent on water availability but also closely related to canopy height, with shorter trees exhibiting an easier recovery. However, the effect of canopy height on forest recovery varied with the water availability, and the better the water availability was, the smaller the effect of canopy height. When the water availability exceeded the threshold of SPEI > 0.85, no significant difference was observed among forests with various heights in their recovery from drought. Considering the increasing frequency and duration of droughts in the context of global climate change, as well as the greater vulnerability and disaster risk faced by tall trees, more in-depth investigations are needed to understand and predict the implications of canopy height for forest management and risk assessment. Based on the results of this study, a number of silvicultural techniques should be explored to reduce the impact of climate change on forests by manipulating the forest structure, specifically by experimentally removing some tall trees that have passed their prime time of carbon fixation with additional risks of mortality or dieback during future drought. This will not only enhance the ecosystem services of forests but also bring economic benefits to the local people in Yunnan.

## Figures and Tables

**Table 1:** The slope (k), coefficient of determination (*R*^2^), and statistical significance (*p* value) of linear regressions between the increase of EVI Deficit (ED) and canopy height, and between enhanced vegetation index (EVI) and canopy height in different forest types.

Forest Type	Height–EVI Increase			Height–ED Increase		
	k	*R* ^2^	*p* Value	k	*R* ^2^	*p* Value
All forest	−0.003	0.76	<0.01	0.466	0.86	<0.01
Needleleaf forest	−0.003	0.65	<0.01	−0.605	0.75	<0.01
Broadleaf forest	−0.004	0.72	<0.01	−0.702	0.81	<0.01

## Supplementary Materials

The following are available online at http://www.mdpi.com/1660-4601/15/6/1257/s1, Figure S1: The percentage of area with moderate or severe drought (SPEI < –1) in the study area during 2001–2014; Figure S2: The relationships between the increase of forest EVI (ED) and canopy height. ED (EVI)-increase represents the ED (EVI) difference between 2012 and 2014 (2014 minus 2012); Figure S3: The relationship between forest recovery and canopy height under various water availability; Figure S4: Changes in SPEI over time under scenarios RCP 2.6 and RCP 8.5 on time scales of 12 months. Table S1: Scale of drought severity based on the standardized precipitation evapotranspiration index (SPEI).
