# Supplementary material for: Impacts of Water Stress on Forest Recovery and Its Interaction with Canopy Height
Source: Int J Environ Res Public Health. 2018 Jun 13;15(6):1257. doi: 10.3390/ijerph15061257 (PMC6025017; doi:10.3390/ijerph15061257)
Supplement: Supplementary file 1 [file ijerph-15-01257-s001.pdf]

SUPPLEMENTARY MATERIAL

Peipei Xu[1,2,3], Tao Zhou[1,2 *], Chuixiang Yi[3], Hui Luo[1,2], Xiang Zhao[4], Shan Gao[1,2], Xia Liu[1,2]

[1] State Key Laboratory of Earth Surface Processes and Resource Ecology, Faculty of Geographical Science, Beijing Normal University, Beijing 100875, China; xupei@mail.bnu.edu.cn.com (PX); Luohui3377@163.com (HL); gsapril@163.com (SG); 201621480027@mail.bnu.edu.cn (XL).

[2] Key Laboratory of Environmental Change and Natural Disaster of Ministry of Education, Academy of Disaster Reduction and Emergency Management, Faculty of Geographical Science, Beijing Normal University, Beijing 100875, China

[3] School of Earth and Environment Science, Queens College of the City University of New York, New York 11367, USA; cyi@qc.cuny.edu.

[4] State Key Laboratory of Remote Sensing Science, Jointly Sponsored by Beijing Normal University and Institute of Remote Sensing and Digital Earth of Chinese Academy of Sciences, Beijing 100875, China; zhaoxiang@bnu.edu.cn.

* Correspondence: tzhou@bnu.edu.cn; Tel.: +86-010-58807238.

Table S1. Scale of drought severity based on the Standardized Precipitation-Evapotranspiration Index (SPEI)

Drought category	SPEI values
extreme drought	SPEI ≤ -2
severe drought	-2 < SPEI ≤ -1.5
moderate drought	-1.5 < SPEI ≤ -1.0
mild drought	-1.0 < SPEI ≤-0.5
Normal	-0.5<SPEI≤0.5
Mild wetness	0.5< SPEI ≤1
moderate wetness	1< SPEI ≤1.5
severe wetness	1.5< SPEI ≤2
extreme wetness	SPEI>2

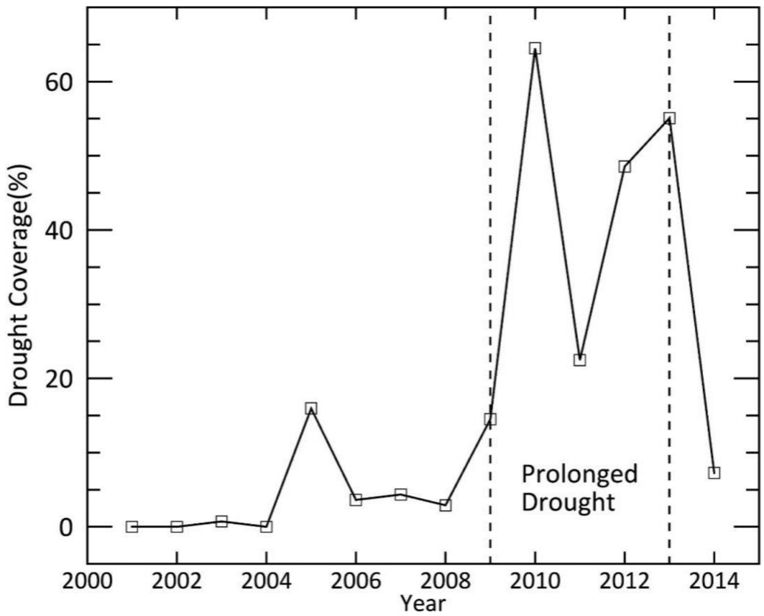

Figure S1. The percentage of area with moderate or severe drought (SPEI < –1) in the study area during 2001–2014.

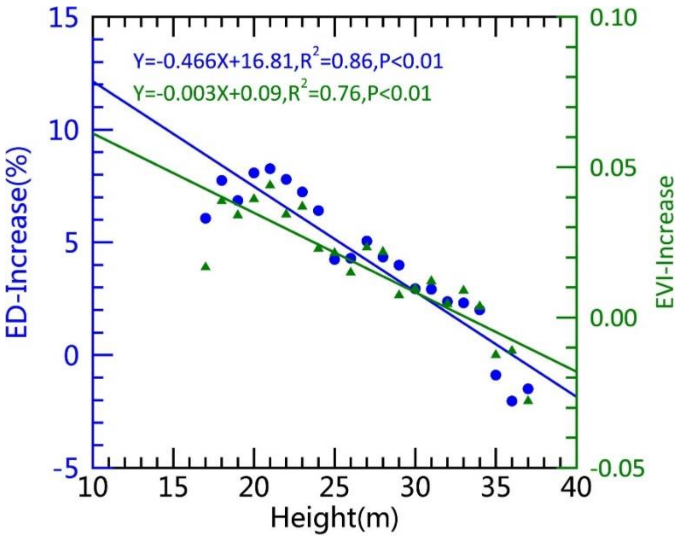

Figure S2. The relationships between the increase of forest EVI (ED) and canopy height. ED (EVI)-Increase represents the ED (EVI) difference between 2012 and 2014 (2014 minus 2012).

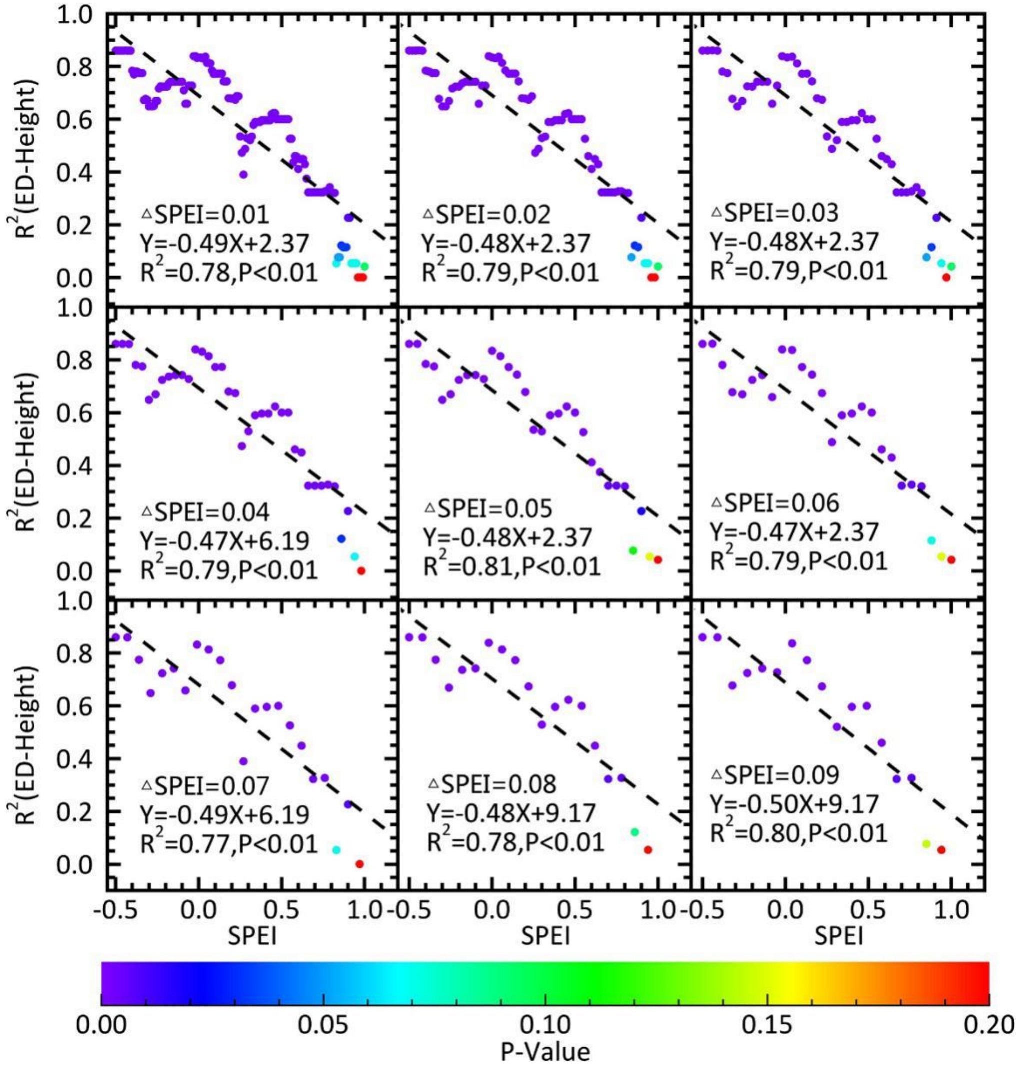

Figure S3. The relationship between forest recovery and canopy height under various water conditions. X-axis represents various water conditions, and Y-axis indicates the determination coefficient (R2) of the regression equation between the increase of ED and canopy height.

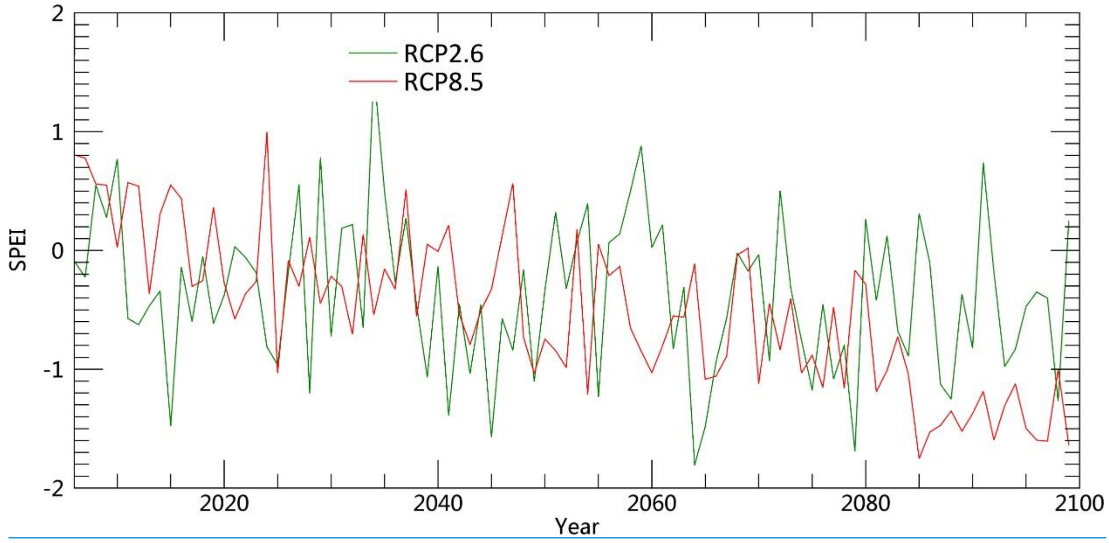

Figure S3 Changes in SPEI over time under scenarios RCP 2.6 and RCP 8.5 on time scales of 12 months
